# Structural, energetic and lipophilic analysis of SARS-CoV-2 non-structural protein 9 (NSP9)

**DOI:** 10.1038/s41598-021-02366-0

**Published:** 2021-11-26

**Authors:** Jéssica de O. Araújo, Silvana Pinheiro, William J. Zamora, Cláudio Nahum Alves, Jerônimo Lameira, Anderson H. Lima

**Affiliations:** 1 Laboratório de Planejamento e Desenvolvimento de Fármacos, Instituto de Ciências Exatas e Naturais, Universidade Federal do Pará, Rua Augusto Corrêa 01, 66075-110 Belém, Pará Brasil; 2 School of Chemistry & Faculty of Pharmacy, University of Costa Rica, San Pedro, San José, Costa Rica; 3 Advanced Computing Lab (CNCA), National High Technology Center (CeNAT-CONARE), Pavas, San José, Costa Rica

**Keywords:** Computational chemistry, Molecular dynamics

## Abstract

In SARS-CoV-2 replication complex, the Non-structural protein 9 (Nsp9) is an important RNA binding subunit in the RNA-synthesizing machinery. The dimeric forms of coronavirus Nsp9 increase their nucleic acid binding affinity and the N-finger motif appears to play a critical role in dimerization. Here, we present a structural, lipophilic and energetic study about the Nsp9 dimer of SARS-CoV-2 through computational methods that complement hydrophobicity scales of amino acids with molecular dynamics simulations. Additionally, we presented a virtual N-finger mutation to investigate whether this motif contributes to dimer stability. The results reveal for the native dimer that the N-finger contributes favorably through hydrogen bond interactions and two amino acids bellowing to the hydrophobic region, Leu45 and Leu106, are crucial in the formation of the cavity for potential drug binding. On the other hand, Gly100 and Gly104, are responsible for stabilizing the α-helices and making the dimer interface remain stable in both, native and mutant (without N-finger motif) systems. Besides, clustering results for the native dimer showed accessible cavities to drugs. In addition, the energetic and lipophilic analysis reveal that the higher binding energy in the native dimer can be deduced since it is more lipophilic than the mutant one, increasing non-polar interactions, which is in line with the result of MM-GBSA and SIE approaches where the van der Waals energy term has the greatest weight in the stability of the native dimer. Overall, we provide a detailed study on the Nsp9 dimer of SARS-CoV-2 that may aid in the development of new strategies for the treatment and prevention of COVID-19.

## Introduction

Severe acute respiratory syndrome coronavirus 2 (SARS-CoV-2) is currently a global pandemic, which has spread rapidly throughout the world since December 2019 when it was first reported^1–6^. During the replication process of the virus, polyprotein processing releases RNA polymerase along with several non-structural proteins (Nsps) that facilitate RNA synthesis and may play a key role in the replication process, although they are not included in the viral envelope^7–11^. All Nsps are considered essential for transcription, replication, and translation of viral RNA, except Nsp1 and Nsp2^2–14^. Nsp9 along with Nsp7, Nsp8, and Nsp10 are located within the replication complex and thus, are likely to be members of this process^12^. In addition, Nsps are considered important for viral replication during the human cell infection phase^13,14^. CoV Nsp9s have diverse forms of dimerization that promote their biological function. SARS-CoV Nsp9 forms a dimer from a conserved region called "GxxxG" α-helical motif, where the interruption of key residues within this region reduces RNA binding and SARS-CoV proliferation^14,15^. Additionally, it was observed that porcine delta coronavirus (PDCoV) Nsp9 mutant (Nsp9 without the N-finger motif) is monomeric in solution^16^. Since the dimeric form of Nsp9 is essential for viral replication and infection, studies suggest that dimer disruption may be an effective strategy in combating coronavirus-associated diseases^13–18^.

Although there is an increasing number of proteins determined by structural techniques as NMR, X-ray diffraction and cryogenic electron microscopy^19,20^ where the formation of protein–protein complexes have evidenced to be essential in biological systems, it is necessary to complement that structural information with a detailed quantitative understanding of the main features that govern the binding mode between the two proteins at an atomic level^21–26^. Accordingly, information about the effect of conformational changes of the two proteins that form dimer species such as lipophilicity, the free energy of binding, movement of the protein's dynamic domain, hot-spot residues in the interaction interface need to be investigated^27–29^.

Experimental evidence has shown through structure and function studies that protein dimerization is controlled by the interaction of hydrophobic surfaces^30^, however, the dynamic, lipophilic, and energetic analysis of protein–protein interactions (PPI) continues to be a major challenge in theoretical studies^31–33^. From a computational point of view, strategies to face these challenges include studies based on lipophylic scales that consider the local context of proteins^34,35^, molecular dynamics simulations and binding free energy calculations, which provide crucial information about the dynamics of complex protein structures and detailed energetic information^36–39^.

We present in this work a structural, lipophilic and energetic study about the Nsp9 dimer of SARS-CoV-2 through computational methods that complement hydrophobicity scales of amino acids with molecular dynamics. To elucidate contacts between residues that make interactions at the dimer interface we have analyzed the impact of structural movements of the dimer already formed, including after the deletion of the N-finger motif. Clustering results led us to find cavities with high druggability scores, placed near hydrophobic residues and accessible to potential drugs. It may aid to the development of new strategies for the treatment and prevention of COVID-19.

## Results and discussion

### Dynamics of Nsp9 native and mutant dimers allows identification of possible binding sites for inhibitors

The non-structural proteins (Nsps) of SARS-CoV-2 are not incorporated into virion particles. Due to their degree of sequence conservation, enzymatic roles, and essentiality of each of the NSPs in SARS-CoV-2, it is believed that these proteins mimic the behavior of homologous proteins in coronaviruses^17^. These Nsps appear to be necessary for viral replication in SARS-CoV and influence pathogenesis^14^. Although they present a close homology among viruses, the interest in Nsps is because they show conserved functions within the life cycle of SARS-Cov-2 that may be susceptible to inhibition^17^.

Non-structural protein 9 (Nsp9) has been considered essential for viral replication during infection of human cells^14^. Several Nsp9 homologs have been identified in many coronaviruses, including SARS-CoV-2. Nsp9 dimerizes via a conserved α-helical motif called “GxxxG”, where disruption of key residues reduces RNA binding and SARS-CoV viral replication^14,15^.

**Figure 1:**
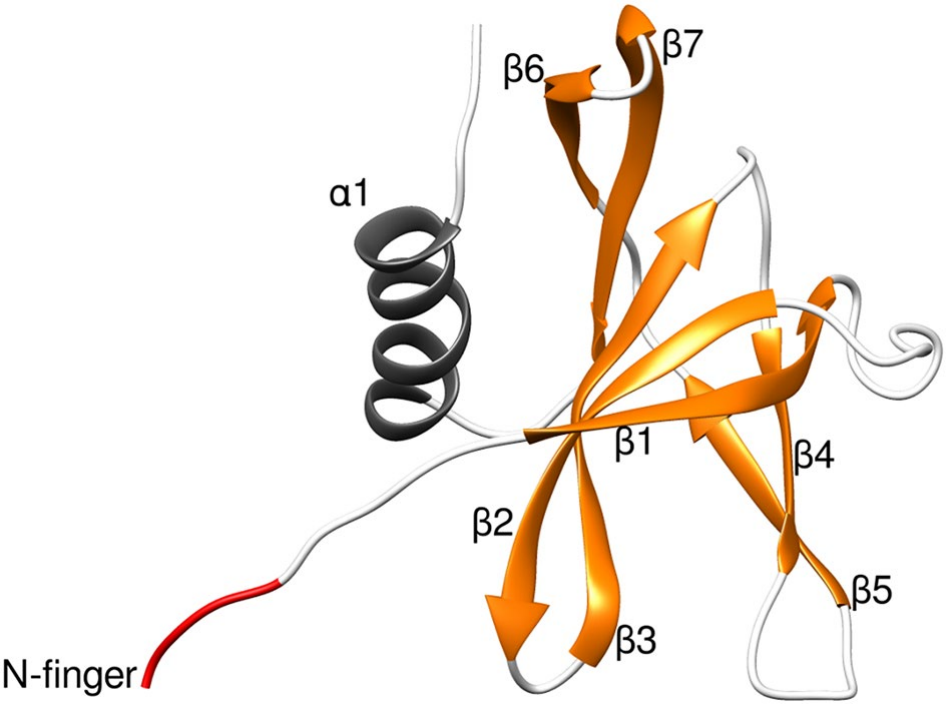
Representation of the monomeric unit of Nsp9. β-sheets are depicted in orange, the α-helix in gray and N-finger motif in red.

The crystal structures of SARS-CoV-2 Nsp9 show an unusual fold seen only in coronaviruses^15,40^. The core of this fold is a small β-barrel enclosed by six β-sheets where a series of extended loops protrude outward. These loops connect to the individual β-sheet of the barrel with an N-terminal β-sheet and a C-terminal α-helix, where the last two elements compose the main regions of the dimer interface (Fig. 1).

Thus, to obtain different conformations of the protein complex and observe how these regions interact in the dimeric interface, 2000 ns of all-atom molecular dynamics simulations were performed. Sampling was obtained for the native and the mutant proteins in order to observe structural, lipophilic, and energetic aspects of these two different systems. It is noteworthy that either the chain A or the chain B in the native Nsp9 dimer present the region called *N-finger* (NNEL residues) at the *N-terminal* region which plays a critical role in the dimerization process^16^. However, the lack of this region in the mutant system imposes a relevant structural difference which can play a crucial role in terms of stability and dynamics.

**Figure 2:**
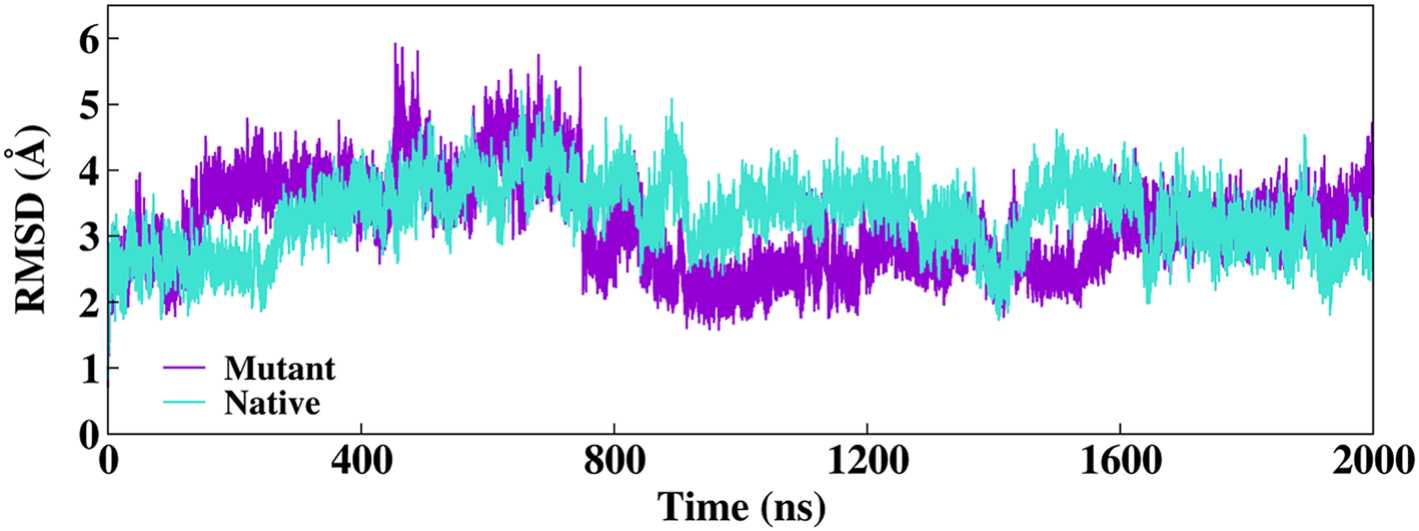
Root mean squared deviation (RMSD, Å) for the protein backbone of each native and mutant system over 2000 ns of MD simulation.

The evolutions of conformations in the systems were analyzed by determining the mean square deviation (RMSD) of each structure with respect to the reference structure of the equilibrium step, which was calculated after alignment based on the backbone atoms (Fig. 2).

**Figure 3:**
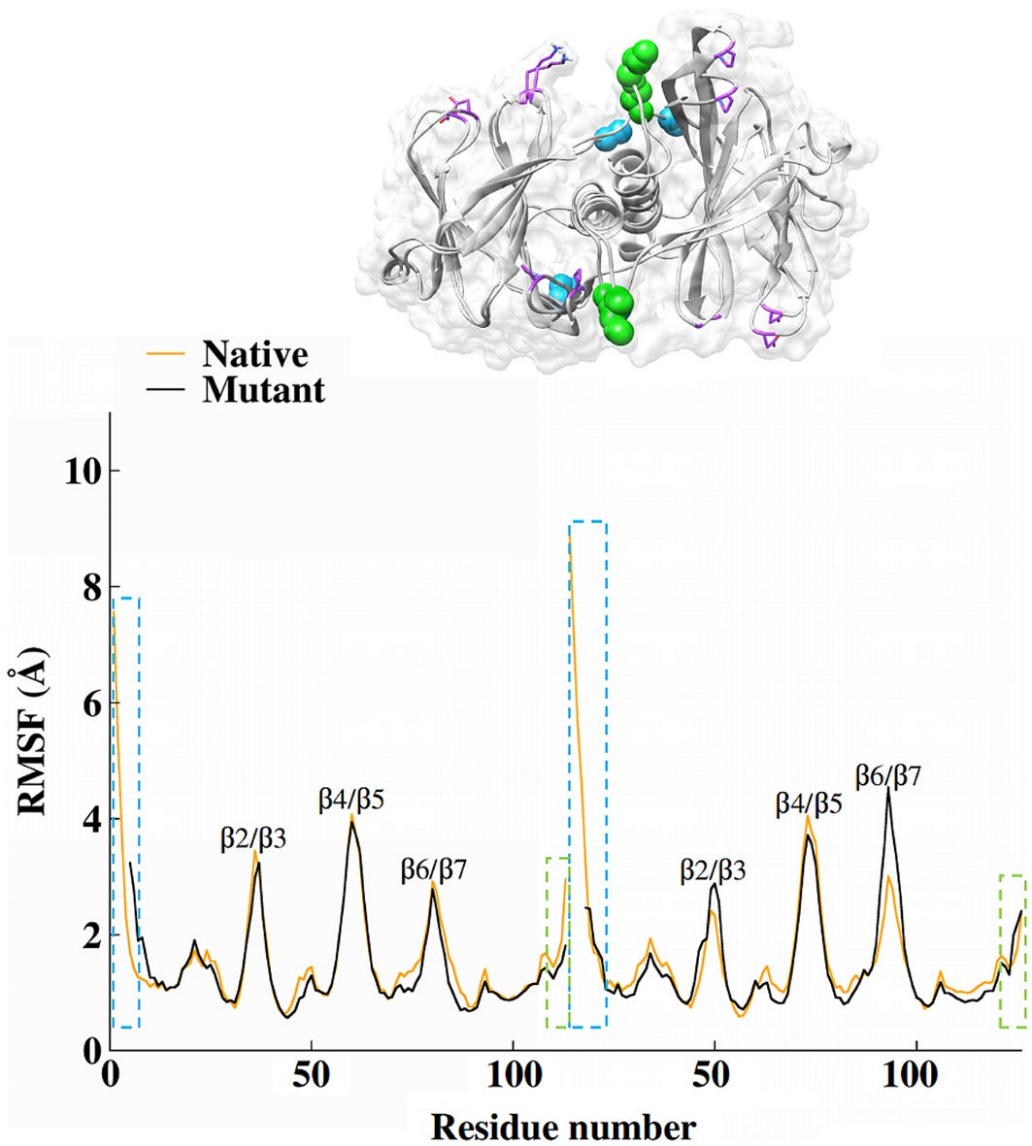
RMSF average of the residue fluctuations obtained along the 800–2000 ns MD simulation for the native (orange) and mutant (black) systems. Highlighting the final structures of the simulation with the N-terminal region (blue), C-terminal green), and residues with high fluctuations in the loops (purple) for both systems.

Figure 2 shows the RMSD for both systems, which was significant stable, particularly after 800 ns of MD simulations where the RMSD values of all systems were within a reasonable fluctuation in a range of 1 to 4 Å suggesting that the structural equilibrium was reached. In addition, visualizations of the sampled structures in the trajectories indicate that some regions of the monomers present moderate movements concerning the initial structure. Comparing the mutant structure against the native as the reference structure, the lDDT score (a local superposition-free score for comparing protein structures) is plotted as a function of the residue numbers. Deviation of no more than 0.9 was observed (Fig. S4). Additionally, the flexibility of each system was verified by means of the fluctuations of the backbone atoms for each residue. In consequence, the Root-Mean-Square Fluctuations (RMSF) were calculated to characterize the local movement of residues in the dimeric systems (see Fig. 3).

Overall, the RMSF data show similar trends obtained for both native and mutant systems. Figure 3 indicates that the residues with higher fluctuation values are located in the N and C-terminals regions (blue and green marks in Fig. 3, respectively), they present high flexibility that amounts to 10 Å. Furthermore, the other regions with relevant fluctuations correspond to regions of the β2/β3, β4/β5, and β6/β7 loops that connect the β-sheets inside the barrel (residues highlighted in purple). The N-terminal regions in monomers A show similar fluctuations, whereas the monomer B in the mutant system has a smaller fluctuation.

**Figure 4:**
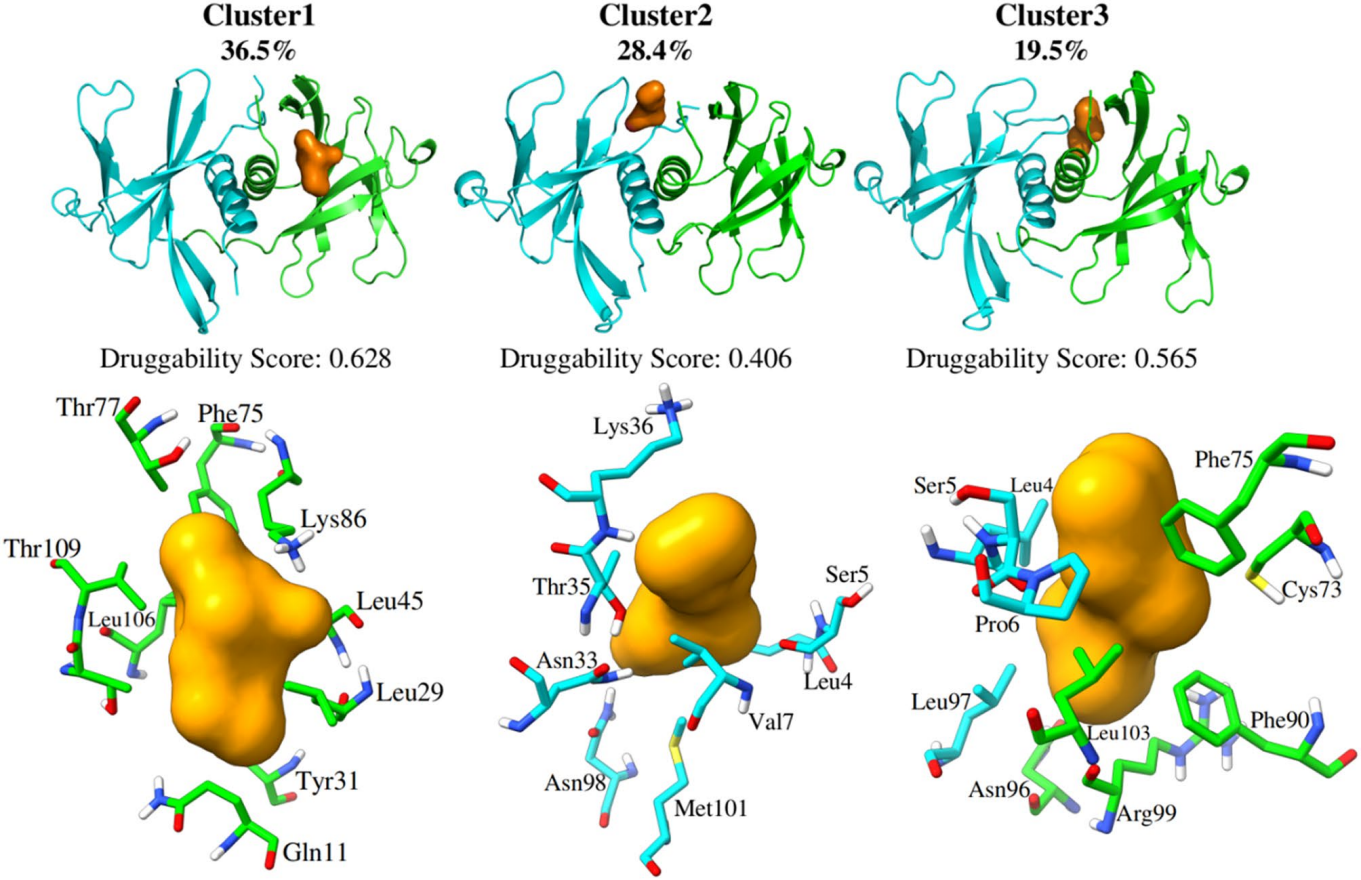
Representative structures of the clustering results for the native dimer showing the accessible cavities (orange surfaces) to drugs calculated with Fpocket. At the bottom, residues that form the cavities are presented in sticks.

To better understand the conformational changes of the binding regions at the dimer interface, it is necessary to analyze the movements in more detail. Therefore, the final trajectories of the native system were analyzed through cluster analysis by grouping the poses extracted from the MD simulations. In general, the three most populated clusters present regions with minor differences in their structure (See Fig. S1 in support information). Following the RMSF analysis (Fig. 3), the loop regions, N and C-terminal present major deviations. Cluster1 showed a conformation similar to cluster3 regarding the loop regions, therefore, there are differences in the terminal regions. In cluster1 the N-terminal of chain A (Fig. 4, in cyan) is interacting with chain B (Fig. 4, in green) which limits cavity formation due to steric impediment. Cluster3 has a cavity located in this region since the N-terminal of chain A is interacting with the same chain and promotes cavity formation nearby the α-helices. On the other hand, Cluster2 presents differences in conformation mainly in the β2/β3 and β3/β4 loops of chain A compared to cluster1 and 2. Thus, its cavity is located closer to cluster3. Figure 4 depicts a representative structure of each of the first 3 dominant clusters, which allowed us to accurately model the structural interfaces of the systems. Additionally, we use these structures with the FPocket software to identify the possible pockets with greater affinity for drugs in the dimer interface regions in each of the selected structures^46^.

Cluster 1 has the highest percentage of structures and is also the one with the highest druggability score, which may help to understand why the cavity formed, where hydrophobic residues Leu45 and Leu106 are present, is the most accessible to possible drugs. These findings are similar to the study by Littler and co-workers who identified the surface of the hydrophobic interface cavity between Nsp9 dimer proteins^17^. This type of cavity analysis^41,42^ has been applied to other protein systems^43^ and has shown promise for screening enzyme inhibitors and may help in the search for molecules with anti-SARS-CoV-2 potential.

**Figure 5:**
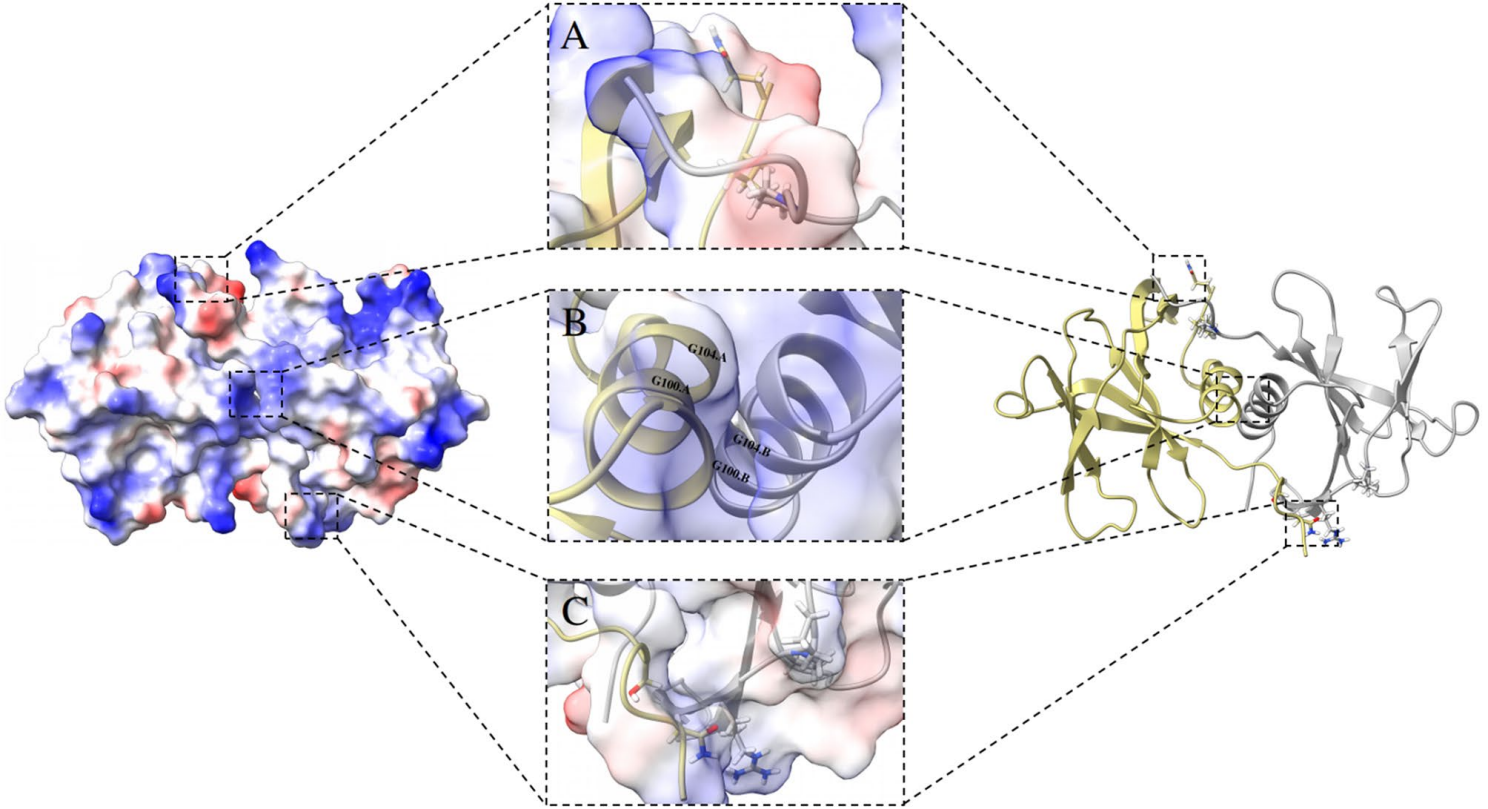
Details of the structure of the dimer with the main regions involved in the contacts between the interfaces. (**A**) and (**C**) represent details of the interactions between the N-terminal region of the monomers. (**B**) shows details of the hydrophobic interactions between the interfaces with residues located near the C-terminal regions of both monomers.

Finally, it is worth mentioning that the N-terminal regions are isolated making contact with counterpart monomer residues (Fig. 5A,C). In contrast, Fig. 5B shows the C-terminal portions surrounded by hydrophobic residues, which causes it to create funnel-shaped hydrophobic cavities on either side of the interface helices.

### Main interactions at the NSP9 dimer interface

It is well known that hydrogen bonds and hydrophobic interactions play important roles in protein–protein interactions^31,44–46^. The arrangement of monomers within Nsp9 dimers is well conserved at different CoVs and is maintained at SARS-CoV-2^17^. The main region of interaction between the monomers is the conserved "GxxxG" protein-binding motif, which allows van der Waals interactions in the interface regions of the C-terminal between the α-helices^47^. The main hydrogen interactions observed at the binding interface of monomers A and B are listed in Table 1, along with its occupancy during the last 100 ns of MD simulations. If there is more than one interaction with the same residues only the highest value is reported.

**Table 1:** Main hydrogen interactions were obtained with the last 100 ns of the MD simulation and their respective occupancies.

**A-B interface**					
Native			Mutant		
A	B	Occupancy (%)	A	B	Occupancy (%)
Asn2	Pro71	51.77	Ser5	Gln226	60.84
Asn2	Gln70	30.80	Gln113	Ser5	18.01
Pro71	Asn2	19.76			
Pro72	Asn2	17.96			
Ser5	Arg74	15.51			

The most stable hydrogen interactions involve residues from the N and C-terminal regions of proteins. Some studies indicate that these regions are important to maintain the structure of the dimer formed^19^. While the native system has a higher number of interactions mainly with residues from the beginning of the chain. The mutant system has only two interactions, one at the beginning and one at the end of the chain. When observing the structures at the end of the simulation, it is possible to notice that the N-finger region located at the N-terminal of monomer A establishes more interactions with the region where the β6 of monomer B is located.

For these systems, the most frequent interactions are van der Waals located in the α-helices, where the residues that contribute to these interactions are mainly Gly100 and Gly104, responsible for stabilizing the α-helices and making the dimer interface remain stable. Our results strongly suggest that these interactions are mainly responsible for the maintenance of the dimeric form of SARs-CoV-2 Nsp9 since experimental data show that the native enzyme with the presence of the N-finger plays an important role in maintaining the stability of the dimer^21^.

### Energetic analysis of NSP9 dimers

In this analysis, we used the last 100 ns trajectories of the MD simulation of the native and mutant systems for protein–protein binding free energy calculations using the MMGBSA and SIE methods (see Table 2). These calculations use a portion of the trajectory from which snapshots were selected. In the case of this study, we used 10,000 frames with an interval equal to 2, resulting in 5000 frames for the calculation.

**Table 2:** Binding free energies for native and mutant systems using the SIE and MM-GBSA approach.

Energy (kcal/mol)	Native	Mutant
∆E_vdw_	− 125.96 ± 8.61	− 79.33 ± 4.82
∆E_ele_	82.16 ± 35.11	152.82 ± 28.33
∆E_surf_	− 14.61 ± 1.08	− 9.20 ± 0.49
∆E_GB_	− 5.10 ± 34.75	− 101.29 ± 26.98
∆G_gas_	− 43.79 ± 36.61	73.48 ± 27.82
∆G_sol_	− 19.71 ± 34.36	− 110.48 ± 26.89
Inter Coulomb	36.53 ± 15.61	67.94 ± 12.60
Reaction Field	− 19.14 ± 14.86	− 57.34 ± 11.11
Cavity	− 21.36 ± 1.35	− 13.62 ± 0.61
Constat	− 2.89	− 2.89
**∆G**_**bind**_** (MMGBSA)**^a^	− **63.51 ± 7.68**	− **36.99 ± 4.25**
**∆G**_**bind**_** (SIE)**^a^	− **16.50 ± 1.04**	− **11.52 ± 0.52**

^a^Computed according to Eqs. (2) and (3) (see “Methods” Section).

Table 2 confirm that the two methods were able to predict a strong binding affinity for the two systems. For the native system, this affinity was − 63.51 kcal/mol, whereas for the mutant system, − 36.99 kcal/mol. Using the SIE method, we were able to describe the same energy trend seen in the MMGBSA method, with values ​​of − 16.50 kcal/mol for the native system and − 11.52 kcal/mol for the mutant system.

**Figure 6:**
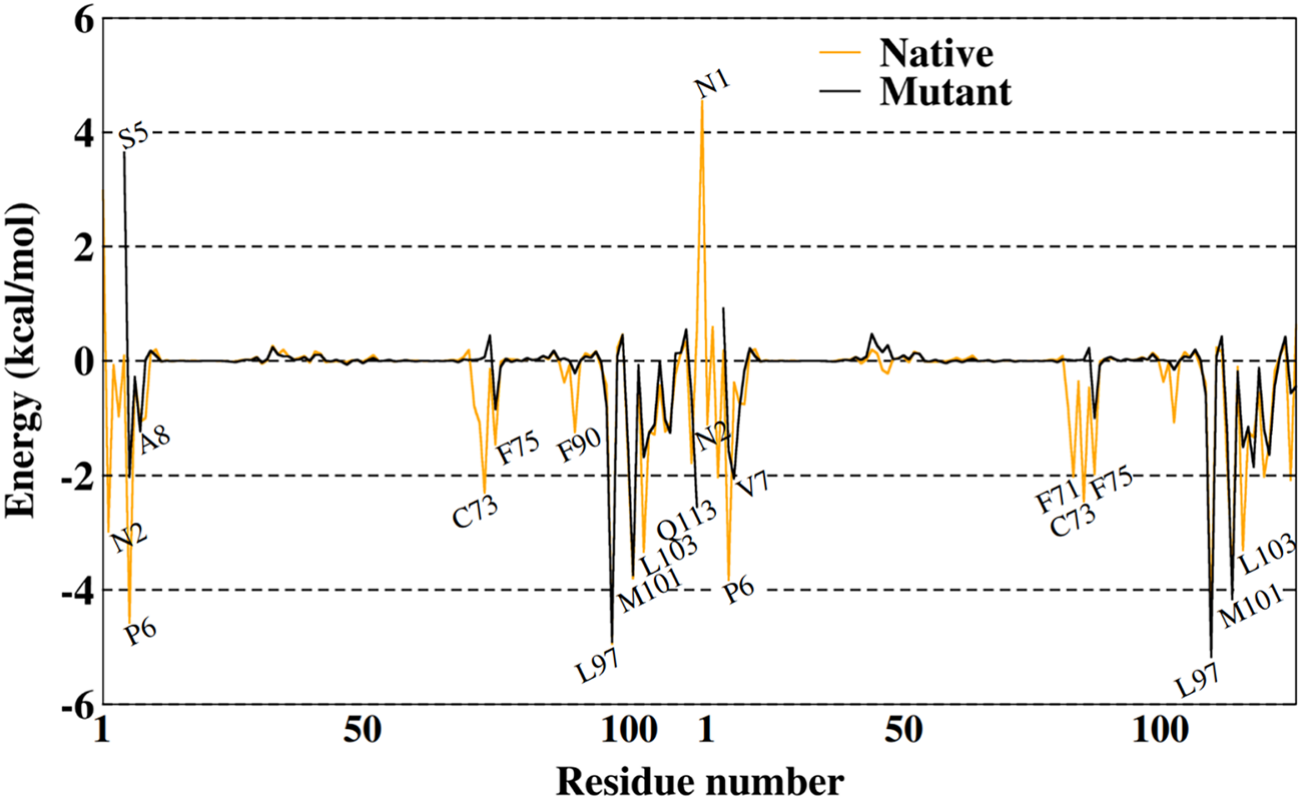
Decomposition of free energy per residue with the MMGBSA method with the energy contribution in terms of a hot spot for the systems.

To identify hot spots of binding affinity between monomers, analyzes of energy decomposition by residue were performed using the MM-GBSA method. The last 100 ns of the trajectories of the two systems were analyzed allowing the description of the residues that are part of the energetic contribution of the protein that assists in the energetic stability process of the dimer. Thus, Fig. 6 shows the contribution of all residues to the binding free energy.

As noted, the residues for both systems show similar peaks. For the native system, the residues located in the N-finger that contribute favorably are residues that are part of the main hydrogen interactions throughout the simulation (see Table 1). On the other hand, the Asn1 residue contributes unfavorably, with positive values near to 2 kcal/mol in monomer A and 4.5 kcal/mol in monomer B.

In this analysis, it was also observed that the fact that the mutant system does not present the N-finger region, the Ser5 residue shows a behavior comparable to Asn1, with an unfavorable contribution. This happens because this region presents high flexibility, making this residue fluctuate during the simulation, establishing few or no interactions.

Residues located in the 97–104 range in both systems contribute favorably to the binding free energy. These are residues found in the contact region of the interface of the monomers where the conserved "GxxxG" motif of Nsp9 is found. Thus, with the decomposition of energy per residue, it is evident that in both systems the main contributions come from residues that are in the interface between the monomers, keeping the dimer stable. It is worth remarking that despite the structural analyses indicate the native system is the one with the greatest movements during the MD simulations, this system is the one with the greatest affinity between the monomers according to the energetic analysis. These observations on predicted binding affinities may be associated with local conformational changes in the N-finger region and in the F71-F75 region, reinforcing that the N-finger region is essential not only for dimer formation but also for maintaining interactions between an interface.

### Analysis of the lipophilicity in NSP9 dimers

**Figure 7:**
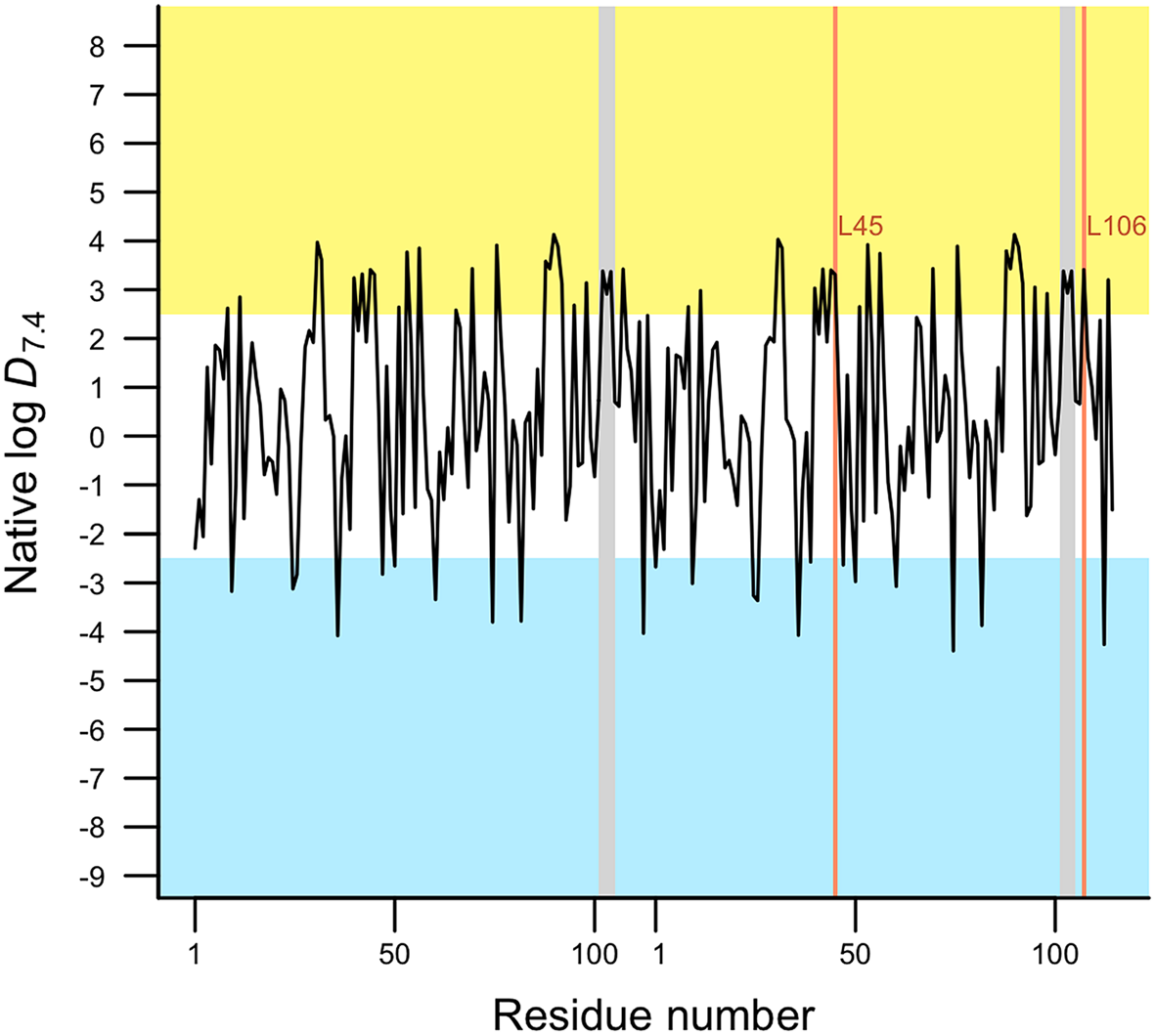
Residue-level lipophilicity expressed as the distribution coefficient of amino acids to pH = 7.4 (log *D*_7.4_) for the native dimer of Nsp9 protein. Highly hydrophobic and hydrophilic regions are shown in yellow and blue sections, respectively. GxxxG motifs are represented in grey rectangles and residues Leu45 and Leu106 in orange lines.

Here, we have complemented the structural and energetic studies with a lipophilic analysis in the native and mutant dimeric forms of Nsp9 protein. This is accomplished by using a novel hydrophobicity scale of amino acids based on quatum-mechanical implicit solvation model. The individual lipophilicity of each amino acid that forms the native dimer of Nsp9 protein is shown in Fig. 7 where hydrophobic residues are present in the yellow region whereas hydrophilic amino acids are in the blue one. In the hydrophobic region, it can be noted that two crucial residues involved in the formation of the cavity for potential drug binding are present, Leu45 and Leu106. These findings support the druggability score in representative structures of the clustering results for the native dimer mentioned above. On the other hand, main interactions at the Nsp9 dimer interface found in this work pointed out the importance of hydrophobic interactions (van der Waals interactions) in the GxxxG protein-binding motif which is in agreement with the lipophilic profile for these fragments (see grey rectangles, Fig. 7) where the residues between glycine residues, M101, V102, and L103, belongs to the highly hydrophobic portions in the dimeric Nsp9 protein.

**Figure 8:**
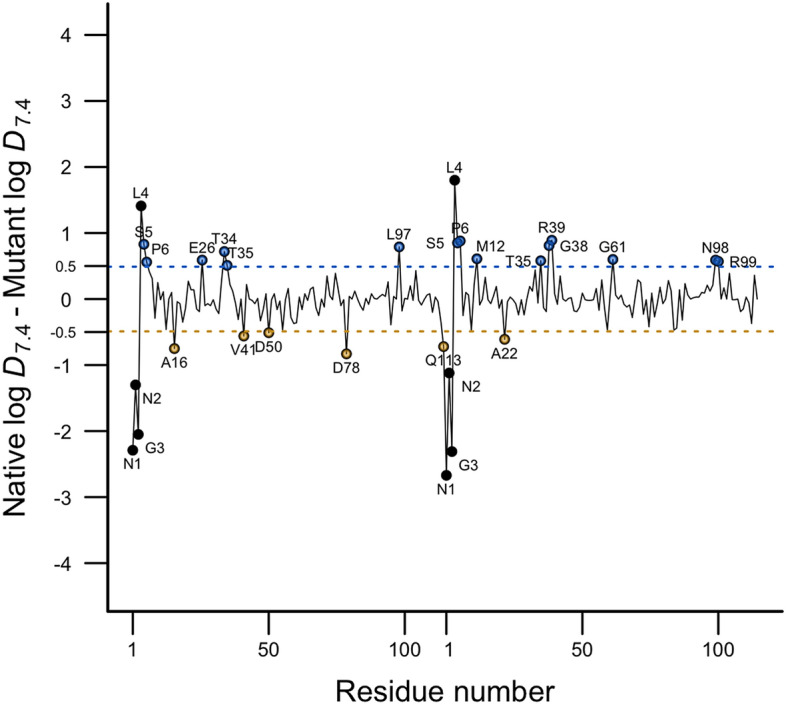
Representation of the difference between cluster-weighted lipophilicities of amino acids in the native form regarding the mutant protein. N-finger residues (black points) and residues whose lipophilicity either decrease (blue points) or increase (yellow points) in the mutant are labeled.

While the previous results showed important structural traits in the native protein, the difference in the cluster-weighted lipophilicities between the native and mutant Nsp9 dimers can provide a counterpart in the energetic analysis of these biomolecules. Figure 8 shows the difference between cluster-weighted residue lipophilicities in the native form regarding the mutant protein where differences higher to 0.5 log units are labeled. Here, a positive difference means that the residue in the mutant concerning the native dimer is more hydrophilic whereas a negative difference implies an increase in the lipophilicity of the amino acid in the mutant. Overall, the hydrophilicity increased by more than 0.5 log units at 17 residues in mutant. Indeed, just in the monomer A (mutant) there is a slightly increase of lipophilicity in some residues (Ala16, Val41, Asp50, Asp78, and Gln113), which is not observed in the monomer B which explains why solvation free energy was the term that most contributes to the stability of the mutant (see Table 2). Accordingly, the higher binding energy in the native dimer can be deduced since it is more lipophilic than the mutant, increasing non-polar interactions, which is in line with the result of MM-GBSA and SIE approaches where the van der Waals energy term has the greatest weight in the stability of the native dimer.

## Conclusions

Herein, we have evaluated structural, energetic and lipophilic aspects of Nsp9 dimers from SARS-CoV-2. The modeled mutant dimer without the N-finger motif revealed that the interaction in the GxxxG protein-binding motif was sufficient to maintain the protein–protein complex bound along with the simulation. This virtual mutation was responsible to decrease the bind of Nsp9 by 26.52 and 4.98 kcal/mol according to MM-GBSA and SIE calculations. The main interactions at the Nsp9 dimer interface found in this work pointed out the importance of hydrophobic interactions (van der Waals interactions) in the GxxxG protein-binding motif which is in agreement with the lipophilic profile. Thus, our results can be useful for understanding why the cavity formed is the most accessible to possible drugs. This type of cavity analysis has been applied to other protein systems and has shown promise for screening enzyme inhibitors and may help in the search for molecules with anti-SARS-CoV-2 potential.

## Materials and methods

### Molecular dynamics simulations of native and mutant systems

Two models were prepared considering both the native and a mutant protein, where the first four residues (NNEL) of N-terminal region are truncated. As a starting point, the crystal structure of Nsp9 RNA binding protein of SARS CoV-2 with 2.95 Å resolution was used (PDB ID 6W4B)^48^. This structure is a dimer in which the N-terminal monomer A needed to be modeled with the *Swiss model*^49^ using monomer B as template. The protonation states in the two systems for all residues were predicted using H++ program at pH 7.0^50,51^. The systems were prepared for molecular dynamics simulations using the AMBER18 package with the force field FF14SB^52,53^. By using the Leap module of Amber^42^ the charges were neutralized by the addition of counterions (Na^+^) and then, the systems were inserted into a cubic box with TIP3P water molecules employing a minimum distance of 12 Å between the protein surface and the side of the box. The models were submitted to four minimization steps before MD simulation. In these four stages, the minimization procedure was applied to the following atoms: First, water molecules and counterions (8000 steps), then, the hydrogen atoms of the protein (5000 steps), next, all hydrogen atoms (8000 steps), and finally, the complete system (10,000 steps).

The models have been submitted to a gradual heating step during 200 ps up to 300 K using a Langevin thermostat at constant volume (NVT ensemble). In the next step of 300 ps the density of the systems was balanced. Then, a total of 500 ps of MD was made with constant pressure to balance the systems before starting MD productions. Finally, the productions were performed by 2000 ns of MD with an NTP ensemble at constant temperature (300 K), using periodic boundary conditions, with a 2 fs integration step using the SHAKE algorithm to restrict bonds involving hydrogen atoms^54,55^. A 10 Å cutoff was used during all the simulations for unconnected interactions. The final trajectories were analyzed in terms of root mean square deviation (RMSD), formation of hydrogen bonds, and cluster analysis. Cluster analyses were performed using the average-linkage hierarchical agglomerative method^45^. For this algorithm the RMSD coordinate was used as a distance metric. The algorithm was used on all heavy atoms of the native (1–226 atoms) and mutant (1–218 atoms) protein with a critical distance value of 3 Å and a variable sieve value to ensure an initial passage of 10 frames through the trajectory^45^. From the cluster analysis in the set of poses, only for the native system was selected a representative structure of each of the three main clusters. In addition, the FPocket software was used to identify possible drug-susceptible pockets in each of the selected structures^42,56^.

### Binding free energy calculations

The protein–protein binding free energy can be expressed according to an MMGBSA approach:^57,58^

1 $$\Delta G_{bind} = < G_{complex} \left( i \right) - G_{protein1} \left( i \right) - G_{protein2} \left( i \right) >$$

where the terms < *Gx* > represent the average over the snapshots of a single trajectory of the MD complex and *i* corresponds to the ith snapshot of the protein complex. AMBER was used to calculate free energy with MMGBSA (Eq. 2) and SIE methods (Eq. 3) for 5000 frames taken from the last 100 ns of MD production^31,53,59^.

2 $$\Delta G_{bind,MMGBSA} = \Delta E_{MM} + \Delta G_{sol} - T\Delta S$$

$\Delta E_{MM}$ is total gas phase energy (sum of $\Delta E_{internal}$, $\Delta E_{electrostatic}$, and $\Delta E_{vdw}$); $\Delta G_{sol}$ is sum of polar $\left( {\Delta G_{GB} } \right)$ and non-polar $\left( {\Delta G_{SA} } \right)$ contributions to solvation.

3 $$\Delta G_{bind,SIE} = \alpha \left[ {E_{C} \left( {D_{in} } \right) + \Delta G_{bind}^{R} \left( {\rho ,D_{in} } \right) + E_{vdw} + \gamma \cdot \Delta MSA\left( \rho \right)} \right] + C$$

$E_{C}$ and $E_{vdw}$ are the intermolecular Coulomb and van der Waals interaction energies in the bound state. $\Delta G_{bind}^{R}$ is the change in the reaction field energy between the bound and free states. The $\Delta MSA$ term is the change in molecular surface area upon binding. The AMBER van der Waals radii linear scaling coefficient (*ρ*), the solute interior dielectric constant $\left( {D_{in} } \right)$, the molecular surface area coefficient (γ), the global proportionality coefficient relating to the loss of configurational entropy upon binding (α), and a constant (*C*) are parameters calibrated by fitting to absolute binding free energies^31^.

As our objective is to analyze the contribution of each energy component and the Gibbs absolute energy, the entropy contribution was not included in the calculations due to the difficulty of accurately calculating entropy for a large protein–protein complex^60^.

In order to identify the main residues responsible for the dimer formation process, free energy decomposition was performed for the contribution of each residue. This contribution was calculated using the decomposition process with MMGBSA in AMBER. All energy components were also calculated for 5000 frames obtained from the last 100 ns of MD production.

### Lipophilicity calculations

The structure-based and pH-dependent lipophilicity scale developed by Zamora et al.^35^ based on the IEFPCM/MST continuum solvation method was employed to determine the lipophilicity of each dimer to pH = 7.4. The lipophilicity of each amino acid was computed using the ProtL scale taking into account its specific structural features in both, native and mutant dimers. ﻿Cluster-weighted lipophilicities of amino acids in the native and mutant proteins were used in this work (see Table S1).

## Supplementary Information

Supplementary Information.
