# Supplementary material for: Structural, energetic and lipophilic analysis of SARS-CoV-2 non-structural protein 9 (NSP9)
Source: Sci Rep. 2021 Nov 26;11:23003. doi: 10.1038/s41598-021-02366-0 (PMC8626507; doi:10.1038/s41598-021-02366-0)
Supplement: Supplementary file 1 — Supplementary Information. [file 41598_2021_2366_MOESM1_ESM.docx]

**SUPPORT INFORMATION**

**Structural, Energetic and Lipophilic Analysis of SARS-CoV-2 Non-Structural Protein 9 (NSP9)**

Jéssica de O. Araújo,^1^ Silvana Pinheiro,^1^ William J. Zamora,^2,3^ Cláudio Nahum Alves,^1^ Jerônimo Lameira,^1^ Anderson H. Lima.^1,*^

^1^Laboratório de Planejamento e Desenvolvimento de Fármacos, Instituto de Ciências Exatas e Naturais, Universidade Federal do Pará, 66075-110, Belém, Pará, Brasil.

^2^School of Chemistry, University of Costa Rica, San Pedro, San José, Costa Rica

^3^Advanced Computing Lab (CNCA), National High Technology Center (CeNAT-CONARE), Pavas, San José, Costa Rica

*Corresponding author: Rua Augusto Corrêa 01. Guamá. Belém - Pará - Brazil. Zipcode: 66075 110. E-mail: anderson@ufpa.br.

**ORCID of the authors:**

Jéssica de O. Araújo: 0000-0003-4968-8005

Cláudio Nahum Alves: 0000-0001-6576-4229

Silvana de S. Pinheiro: 0000-0002-6909-1129

William J. Zamora: 0000-0003-4029-4528

Jerônimo Lameira: 0000-0001-7270-1517

Anderson H. Lima: 0000-0002-8451-9912


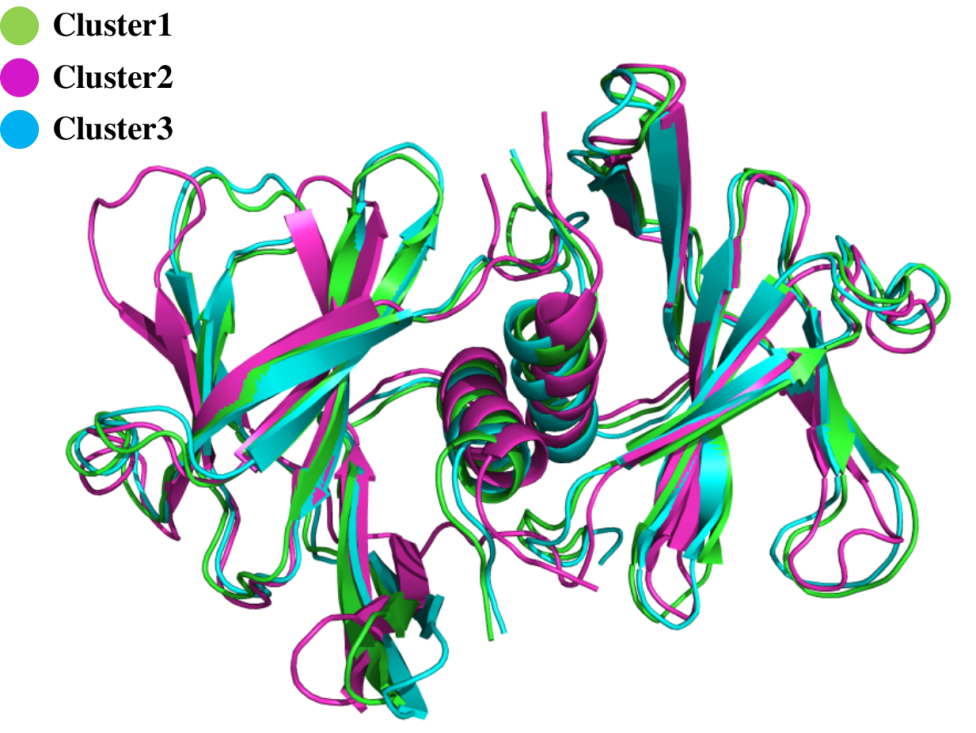


**Figure S1.** Superimposition of the centroids structures from the three most populated clusters.


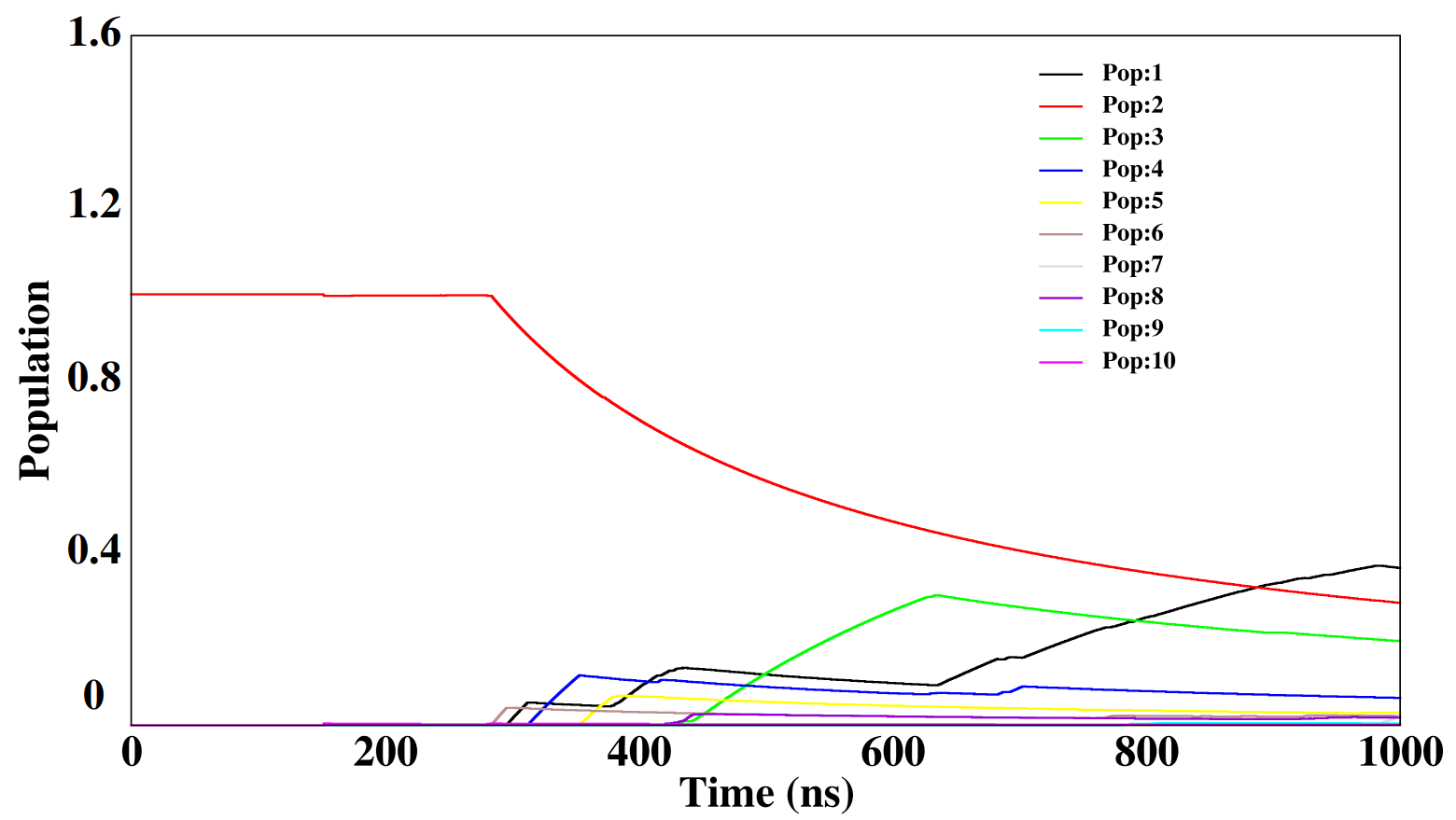


**Figure S2.** Population versus time (ns) of the clusters computed by hierarchical method.


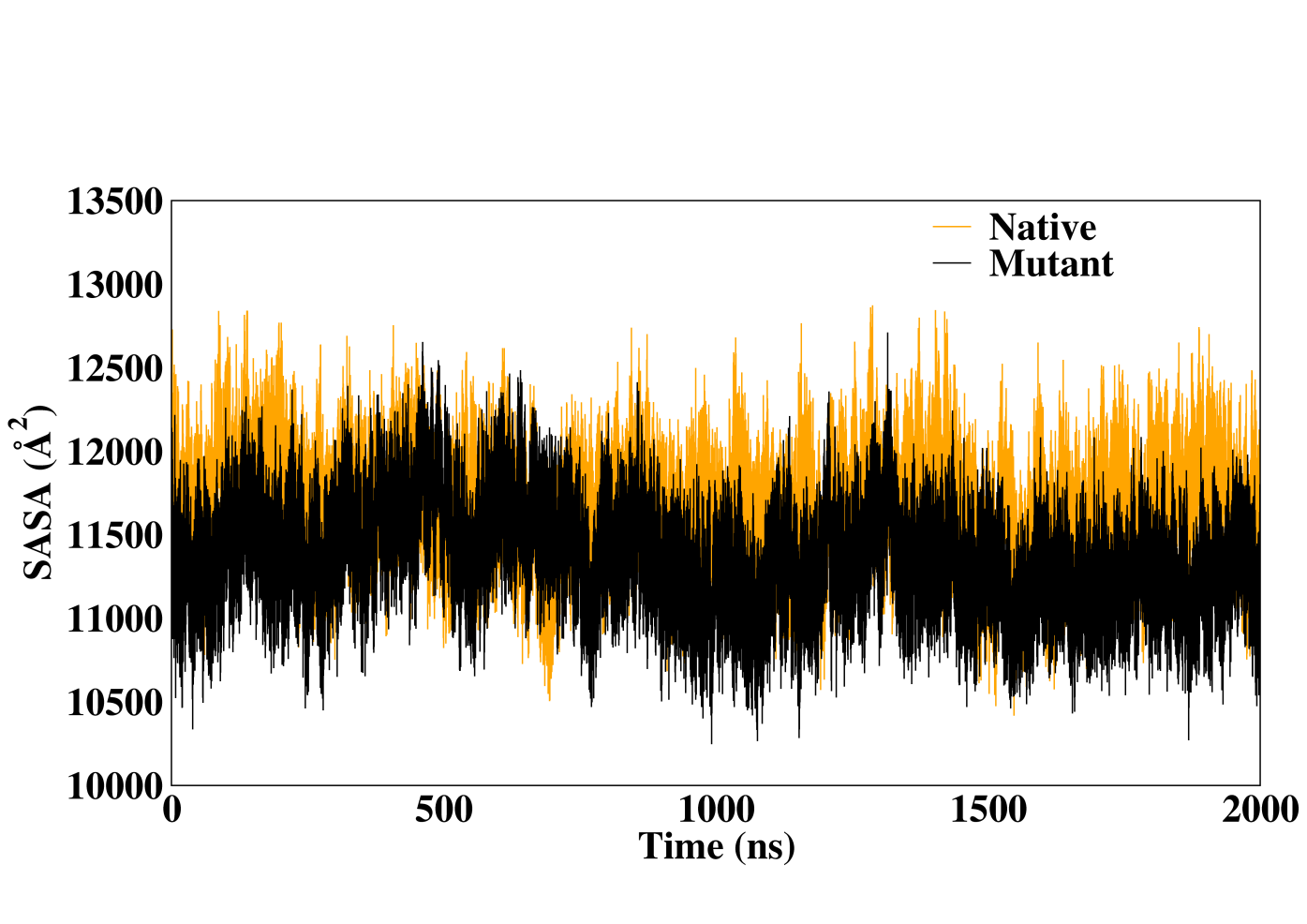


**Figure S3.** Solvent accessible surface area vs. simulation time.

**
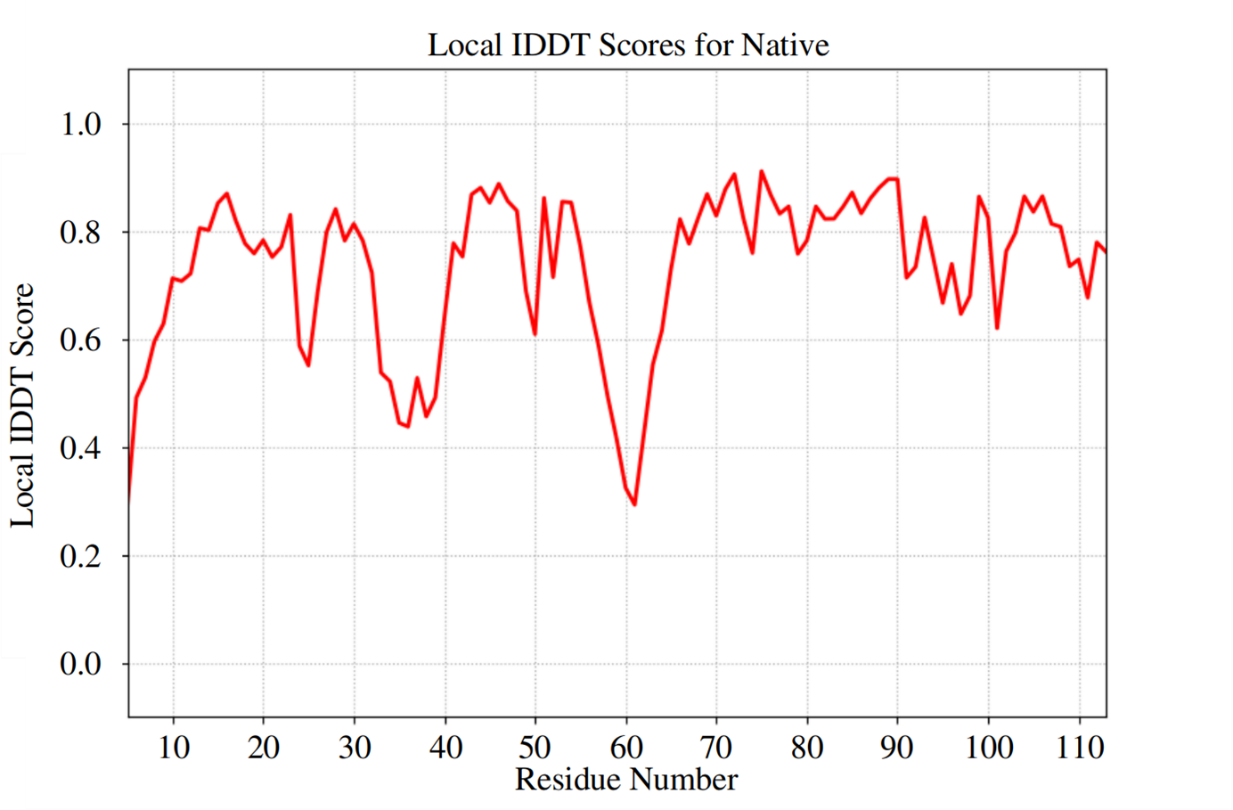
**

**Figure S4.** Local superposition-free score for comparing structures of Nsp9 wild-type and mutant. RMSD of the common residues = 10.935. TM-score = 0.2453 (d0= 3.92).

**Table S1.** Cluster-weighted lipophilicities of amino acids in the native and mutant proteins.

| **id** | **res** | **NATIVE** | | | | **MUTANT** | | | |
| --- | --- | --- | --- | --- | --- | --- | --- | --- | --- |
|  |  | **cluster1 (43.2 %)** | **cluster2 (33.6 %)** | **cluster3 (23.2 %)** | **w.average** | **cluster1 (60.2 %)** | **cluster2 (20.3 %)** | **cluster3 (19.5 %)** | **w.average** |
| 1 | ASN | -2,34 | -2,25 | -2,28 | **-2,29** | - | - | - | **-** |
| 2 | ASN | -0,45 | -2,48 | -1,16 | **-1,30** | - | - | - | **-** |
| 3 | GLU | -3,27 | -0,88 | -1,47 | **-2,05** | - | - | - | **-** |
| 4 | LEU | 2,13 | 1,46 | -0,01 | **1,41** | - | - | - | **-** |
| 5 | SER | 0,18 | -1,13 | -1,16 | **-0,57** | -1,76 | -0,56 | -1,16 | **-1,40** |
| 6 | PRO | 1,88 | 1,89 | 1,79 | **1,86** | 1,64 | 0,53 | 1,04 | **1,30** |
| 7 | VAL | 1,71 | 1,54 | 2,18 | **1,76** | 0,45 | 2,77 | 2,64 | **1,35** |
| 8 | ALA | 1,07 | 1,14 | 1,39 | **1,17** | 0,74 | 1,29 | 0,80 | **0,86** |
| 9 | LEU | 2,54 | 2,70 | 2,67 | **2,62** | 2,62 | 3,32 | 3,38 | **2,91** |
| 10 | ARG | -3,27 | -3,41 | -2,63 | **-3,17** | -3,63 | -3,48 | -2,70 | **-3,42** |
| 11 | GLN | -1,36 | -0,68 | -0,82 | **-1,01** | -0,98 | -0,90 | -1,15 | **-1,00** |
| 12 | MET | 2,65 | 3,01 | 3,00 | **2,85** | 2,99 | 2,24 | 2,47 | **2,74** |
| 13 | SER | -1,68 | -1,57 | -1,89 | **-1,69** | -1,19 | -1,25 | -1,33 | **-1,23** |
| 14 | CYS | 0,73 | 0,73 | 0,73 | **0,73** | 0,73 | 0,73 | 0,73 | **0,73** |
| 15 | ALA | 1,92 | 1,92 | 1,87 | **1,91** | 1,78 | 1,92 | 1,80 | **1,81** |
| 16 | ALA | 1,19 | 1,19 | 1,16 | **1,18** | 1,93 | 1,93 | 1,93 | **1,93** |
| 17 | GLY | 0,65 | 0,56 | 0,60 | **0,61** | 0,63 | 0,68 | 0,66 | **0,65** |
| 18 | THR | -0,71 | -0,90 | -0,78 | **-0,79** | -0,76 | -0,76 | -0,58 | **-0,72** |
| 19 | THR | -0,71 | 0,15 | -0,78 | **-0,44** | 0,07 | 0,03 | -0,70 | **-0,09** |
| 20 | GLN | -0,25 | -1,06 | -0,28 | **-0,53** | -0,25 | -0,23 | -1,01 | **-0,39** |
| 21 | THR | -1,20 | -1,18 | -1,21 | **-1,19** | -1,46 | -1,44 | -1,46 | **-1,46** |
| 22 | ALA | 1,09 | 0,95 | 0,75 | **0,96** | 0,87 | 1,11 | 0,34 | **0,82** |
| 23 | CYS | 0,73 | 0,73 | 0,73 | **0,73** | 0,73 | 0,73 | 0,00 | **0,59** |
| 24 | THR | -0,28 | -0,08 | -0,42 | **-0,24** | -0,13 | -0,07 | 0,03 | **-0,09** |
| 25 | ASP | -3,35 | -3,35 | -2,36 | **-3,12** | -2,93 | -2,98 | -2,87 | **-2,93** |
| 26 | ASP | -2,66 | -2,71 | -3,35 | **-2,83** | -3,35 | -3,78 | -3,25 | **-3,42** |
| 27 | ASN | -0,20 | -0,23 | 0,05 | **-0,15** | -0,06 | 0,05 | -0,12 | **-0,05** |
| 28 | ALA | 1,83 | 1,85 | 1,78 | **1,82** | 1,89 | 1,88 | 1,90 | **1,89** |
| 29 | LEU | 2,16 | 2,05 | 2,32 | **2,16** | 2,27 | 2,32 | 2,19 | **2,26** |
| 30 | ALA | 1,90 | 1,93 | 1,93 | **1,92** | 1,93 | 1,93 | 1,93 | **1,93** |
| 31 | TYR | 3,79 | 4,10 | 4,13 | **3,97** | 4,13 | 4,10 | 4,10 | **4,12** |
| 32 | TYR | 3,79 | 3,44 | 3,59 | **3,62** | 3,79 | 4,10 | 3,72 | **3,84** |
| 33 | ASN | 0,25 | 0,50 | 0,25 | **0,33** | -0,14 | 0,42 | 0,25 | **0,05** |
| 34 | THR | 0,50 | 0,35 | 0,36 | **0,42** | -0,48 | 0,43 | -0,48 | **-0,30** |
| 35 | THR | 0,15 | 0,13 | -0,51 | **-0,01** | -0,91 | 0,15 | 0,00 | **-0,52** |
| 36 | LYS | -4,12 | -4,31 | -3,70 | **-4,08** | -4,17 | -4,36 | -4,65 | **-4,30** |
| 37 | GLY | -1,46 | -0,66 | -0,09 | **-0,87** | -1,28 | -0,13 | -0,97 | **-0,99** |
| 38 | GLY | 0,07 | 0,48 | -0,81 | **0,00** | -0,24 | 0,60 | 0,43 | **0,06** |
| 39 | ARG | -1,71 | -2,21 | -1,86 | **-1,91** | -1,41 | -1,05 | -2,78 | **-1,60** |
| 40 | PHE | 3,25 | 3,09 | 3,45 | **3,24** | 3,25 | 3,13 | 3,32 | **3,24** |
| 41 | VAL | 2,14 | 2,16 | 2,20 | **2,16** | 2,91 | 2,83 | 2,04 | **2,72** |
| 42 | LEU | 3,40 | 3,19 | 3,38 | **3,32** | 2,97 | 3,40 | 3,19 | **3,10** |
| 43 | ALA | 1,93 | 1,93 | 1,93 | **1,93** | 1,93 | 1,93 | 1,93 | **1,93** |
| 44 | LEU | 3,40 | 3,43 | 3,43 | **3,41** | 3,43 | 3,43 | 3,43 | **3,43** |
| 45 | LEU | 3,35 | 3,35 | 3,21 | **3,31** | 3,40 | 3,32 | 3,38 | **3,38** |
| 46 | SER | 0,67 | 0,64 | 0,67 | **0,66** | 0,67 | 0,61 | 0,64 | **0,65** |
| 47 | ASP | -3,14 | -2,87 | -2,18 | **-2,82** | -2,61 | -2,02 | -2,61 | **-2,49** |
| 48 | LEU | 1,35 | 1,49 | 1,49 | **1,43** | 1,78 | 1,13 | 1,49 | **1,59** |
| 49 | GLN | -1,31 | -1,67 | -1,61 | **-1,50** | -1,64 | -1,39 | -1,58 | **-1,58** |
| 50 | ASP | -2,77 | -2,71 | -2,34 | **-2,65** | -1,81 | -2,71 | -2,55 | **-2,14** |
| 51 | LEU | 2,65 | 2,67 | 2,59 | **2,64** | 2,67 | 2,59 | 2,65 | **2,65** |
| 52 | LYS | -1,73 | -1,40 | -1,59 | **-1,59** | -1,59 | -1,69 | -1,88 | **-1,67** |
| 53 | TRP | 3,59 | 3,94 | 3,86 | **3,77** | 3,98 | 3,77 | 3,92 | **3,93** |
| 54 | ALA | 1,86 | 1,93 | 1,93 | **1,90** | 1,93 | 1,93 | 1,93 | **1,93** |
| 55 | ARG | -1,55 | -1,48 | -1,27 | **-1,46** | -1,05 | -0,48 | -1,34 | **-0,99** |
| 56 | PHE | 3,81 | 3,90 | 3,85 | **3,85** | 3,87 | 3,90 | 3,77 | **3,86** |
| 57 | PRO | 0,49 | 1,24 | 0,98 | **0,85** | 0,58 | 0,92 | 0,77 | **0,69** |
| 58 | LYS | -0,93 | -1,40 | -0,88 | **-1,08** | -0,64 | -0,83 | -1,26 | **-0,80** |
| 59 | SER | -1,80 | -0,41 | -1,71 | **-1,31** | -0,78 | -1,48 | -0,87 | **-0,94** |
| 60 | ASP | -2,82 | -4,26 | -2,98 | **-3,34** | -2,93 | -2,98 | -3,14 | **-2,98** |
| 61 | GLY | -0,29 | -0,50 | -0,18 | **-0,33** | -0,18 | -1,29 | 0,06 | **-0,36** |
| 62 | THR | -1,16 | -1,53 | -1,23 | **-1,30** | -1,15 | -1,05 | -1,20 | **-1,14** |
| 63 | GLY | 0,14 | 0,14 | 0,26 | **0,17** | 0,09 | -0,03 | 0,21 | **0,09** |
| 64 | THR | -0,76 | -0,85 | -0,68 | **-0,77** | -0,68 | -0,80 | -0,90 | **-0,75** |
| 65 | ILE | 2,60 | 2,65 | 2,45 | **2,58** | 2,47 | 2,29 | 2,43 | **2,43** |
| 66 | TYR | 2,33 | 2,03 | 2,35 | **2,23** | 1,98 | 2,13 | 2,17 | **2,05** |
| 67 | THR | 0,36 | 0,31 | 0,48 | **0,37** | 0,58 | 0,30 | 0,48 | **0,50** |
| 68 | GLU | -1,72 | -0,07 | -1,24 | **-1,05** | -0,74 | -0,78 | -1,03 | **-0,80** |
| 69 | LEU | 3,43 | 3,43 | 3,43 | **3,43** | 3,43 | 3,43 | 3,43 | **3,43** |
| 70 | GLU | 0,06 | -0,90 | -0,11 | **-0,30** | -0,07 | -0,11 | -0,61 | **-0,18** |
| 71 | PRO | 0,68 | -0,13 | -0,11 | **0,22** | -0,13 | -0,13 | -0,14 | **-0,13** |
| 72 | PRO | 1,37 | 1,26 | 1,24 | **1,30** | 1,14 | 1,36 | 1,47 | **1,25** |
| 73 | CYS | 0,73 | 0,73 | 0,73 | **0,73** | 0,74 | 0,74 | 0,74 | **0,74** |
| 74 | ARG | -3,43 | -4,00 | -4,21 | **-3,80** | -4,28 | -4,36 | -3,71 | **-4,19** |
| 75 | PHE | 3,97 | 3,82 | 3,93 | **3,91** | 3,75 | 3,68 | 3,85 | **3,76** |
| 76 | VAL | 2,40 | 2,37 | 0,82 | **2,02** | 2,16 | 2,24 | 1,86 | **2,12** |
| 77 | THR | 0,66 | 0,65 | 0,61 | **0,64** | 0,63 | 0,65 | 0,70 | **0,65** |
| 78 | ASP | -1,56 | -0,92 | -3,35 | **-1,76** | -0,97 | -0,92 | -0,81 | **-0,93** |
| 79 | THR | 0,31 | 0,31 | 0,36 | **0,32** | 0,27 | 0,27 | 0,33 | **0,28** |
| 80 | PRO | -0,17 | -0,19 | -0,17 | **-0,18** | -0,18 | -0,18 | -0,18 | **-0,18** |
| 81 | LYS | -3,84 | -3,98 | -3,41 | **-3,78** | -3,70 | -4,27 | -4,12 | **-3,90** |
| 82 | GLY | 0,29 | 0,26 | 0,23 | **0,27** | 0,26 | 0,23 | 0,33 | **0,27** |
| 83 | PRO | 0,74 | 0,57 | -0,13 | **0,48** | 0,53 | 0,69 | 0,61 | **0,58** |
| 84 | LYS | -1,69 | -1,59 | -0,98 | **-1,49** | -1,31 | -1,17 | -1,50 | **-1,32** |
| 85 | VAL | 1,33 | 1,56 | 1,19 | **1,37** | 1,40 | 1,20 | 1,40 | **1,36** |
| 86 | LYS | -0,31 | -0,60 | -0,22 | **-0,39** | -0,55 | 0,16 | -0,08 | **-0,31** |
| 87 | TYR | 3,50 | 3,53 | 3,81 | **3,58** | 3,32 | 3,79 | 3,75 | **3,50** |
| 88 | LEU | 3,43 | 3,43 | 3,43 | **3,43** | 3,43 | 3,38 | 3,43 | **3,42** |
| 89 | TYR | 4,13 | 4,13 | 4,13 | **4,13** | 4,13 | 4,13 | 4,13 | **4,13** |
| 90 | PHE | 3,91 | 3,86 | 3,92 | **3,89** | 3,84 | 3,84 | 3,87 | **3,85** |
| 91 | ILE | 3,11 | 3,09 | 3,21 | **3,12** | 3,00 | 3,21 | 3,04 | **3,05** |
| 92 | LYS | -1,90 | -1,78 | -1,31 | **-1,72** | -1,69 | -1,50 | -2,26 | **-1,76** |
| 93 | GLY | -1,36 | -0,60 | -1,06 | **-1,03** | -1,19 | -1,46 | -1,44 | **-1,29** |
| 94 | LEU | 2,35 | 3,13 | 2,67 | **2,68** | 3,21 | 2,46 | 3,27 | **3,07** |
| 95 | ASN | -0,65 | -0,68 | -0,43 | **-0,61** | -0,54 | -0,85 | -0,76 | **-0,65** |
| 96 | ASN | -0,65 | -0,34 | -0,68 | **-0,55** | -0,62 | -0,43 | -0,45 | **-0,55** |
| 97 | LEU | 3,21 | 3,13 | 3,02 | **3,14** | 2,19 | 2,57 | 2,62 | **2,35** |
| 98 | ASN | -0,59 | 0,28 | 0,67 | **-0,01** | 0,14 | 0,34 | 0,11 | **0,17** |
| 99 | ARG | -0,48 | -1,48 | -0,56 | **-0,83** | -0,56 | -1,41 | -1,13 | **-0,84** |
| 100 | GLY | 0,73 | 0,73 | 0,73 | **0,73** | 0,73 | 0,73 | 0,73 | **0,73** |
| 101 | MET | 3,25 | 3,44 | 3,53 | **3,38** | 3,09 | 3,47 | 3,14 | **3,18** |
| 102 | VAL | 2,88 | 2,93 | 2,93 | **2,91** | 2,93 | 2,93 | 2,93 | **2,93** |
| 103 | LEU | 3,38 | 3,35 | 3,40 | **3,37** | 2,97 | 2,78 | 3,02 | **2,94** |
| 104 | GLY | 0,73 | 0,65 | 0,73 | **0,70** | 0,73 | 0,71 | 0,71 | **0,72** |
| 105 | SER | 0,50 | 0,73 | 0,64 | **0,61** | 0,73 | 0,67 | 0,64 | **0,70** |
| 106 | LEU | 3,43 | 3,40 | 3,43 | **3,42** | 3,43 | 3,43 | 3,43 | **3,43** |
| 107 | ALA | 1,84 | 1,75 | 1,70 | **1,78** | 1,76 | 1,76 | 1,58 | **1,72** |
| 108 | ALA | 1,35 | 1,52 | 0,98 | **1,32** | 1,32 | 1,67 | 0,53 | **1,24** |
| 109 | THR | -0,25 | -0,03 | 0,05 | **-0,11** | 0,12 | 0,10 | 0,00 | **0,09** |
| 110 | VAL | 2,29 | 2,38 | 2,40 | **2,34** | 2,35 | 2,09 | 2,42 | **2,31** |
| 111 | ARG | -3,84 | -4,27 | -4,07 | **-4,03** | -3,91 | -4,05 | -4,20 | **-3,99** |
| 112 | LEU | 2,40 | 2,51 | 2,54 | **2,47** | 2,65 | 3,35 | 2,67 | **2,80** |
| 113 | GLN | -0,91 | -1,29 | -1,47 | **-1,17** | -0,31 | -0,58 | -0,77 | **-0,45** |
| 1 | ASN | -3,52 | -2,93 | -0,71 | **-2,67** | - | - | - | **-** |
| 2 | ASN | -1,49 | 0,03 | -2,09 | **-1,12** | - | - | - | **-** |
| 3 | GLU | -0,63 | -4,02 | -2,97 | **-2,31** | - | - | - | **-** |
| 4 | LEU | -0,01 | 3,11 | 3,30 | **1,80** | - | - | - | **-** |
| 5 | SER | -1,39 | -0,84 | -0,99 | **-1,11** | -1,86 | -2,61 | -1,59 | **-1,96** |
| 6 | PRO | 1,89 | 1,22 | 1,88 | **1,66** | 0,88 | 0,75 | 0,52 | **0,78** |
| 7 | VAL | 1,80 | 1,31 | 1,69 | **1,61** | 1,75 | 2,11 | 1,94 | **1,86** |
| 8 | ALA | 1,13 | 0,16 | 1,90 | **0,98** | 1,05 | 0,93 | 0,29 | **0,88** |
| 9 | LEU | 2,67 | 2,65 | 2,62 | **2,65** | 2,59 | 2,51 | 2,65 | **2,59** |
| 10 | ARG | -3,20 | -3,34 | -2,20 | **-3,01** | -2,20 | -2,55 | -3,55 | **-2,53** |
| 11 | GLN | -1,28 | -1,17 | -0,90 | **-1,15** | -1,45 | -1,58 | -0,85 | **-1,36** |
| 12 | MET | 3,02 | 2,97 | 2,94 | **2,98** | 2,33 | 2,74 | 2,11 | **2,37** |
| 13 | SER | -1,07 | -1,48 | -1,65 | **-1,34** | -1,16 | -1,42 | -1,33 | **-1,25** |
| 14 | CYS | 0,73 | 0,73 | 0,73 | **0,73** | 0,73 | 0,73 | 0,73 | **0,73** |
| 15 | ALA | 1,78 | 1,71 | 1,83 | **1,77** | 1,44 | 1,81 | 1,04 | **1,44** |
| 16 | ALA | 1,93 | 1,92 | 1,93 | **1,92** | 1,93 | 1,93 | 1,93 | **1,93** |
| 17 | GLY | 0,61 | 0,61 | 0,66 | **0,62** | 0,70 | 0,65 | 0,68 | **0,69** |
| 18 | THR | -0,58 | -0,75 | -0,65 | **-0,65** | -0,66 | -0,73 | -0,81 | **-0,70** |
| 19 | THR | -0,75 | -0,05 | -0,70 | **-0,50** | -0,71 | -0,63 | -0,71 | **-0,69** |
| 20 | GLN | -1,23 | -0,31 | -1,12 | **-0,89** | -1,04 | -0,33 | -0,31 | **-0,75** |
| 21 | THR | -1,43 | -1,41 | -1,43 | **-1,42** | -1,41 | -1,39 | -1,44 | **-1,41** |
| 22 | ALA | 0,24 | 0,86 | 0,06 | **0,41** | 1,09 | 1,14 | 0,70 | **1,02** |
| 23 | CYS | 0,00 | 0,73 | 0,00 | **0,25** | 0,00 | 0,73 | 0,73 | **0,29** |
| 24 | THR | -0,15 | -0,12 | -0,05 | **-0,12** | -0,15 | -0,18 | -0,12 | **-0,15** |
| 25 | ASP | -3,40 | -3,51 | -2,66 | **-3,26** | -3,56 | -2,87 | -2,71 | **-3,25** |
| 26 | ASP | -3,14 | -3,35 | -3,78 | **-3,36** | -3,19 | -3,72 | -3,14 | **-3,29** |
| 27 | ASN | -0,62 | 0,08 | 0,05 | **-0,23** | 0,03 | 0,00 | -0,06 | **0,01** |
| 28 | ALA | 1,89 | 1,84 | 1,85 | **1,86** | 1,88 | 1,88 | 1,84 | **1,87** |
| 29 | LEU | 1,64 | 2,30 | 2,32 | **2,02** | 2,27 | 2,19 | 2,32 | **2,26** |
| 30 | ALA | 1,93 | 1,93 | 1,93 | **1,93** | 1,93 | 1,93 | 1,93 | **1,93** |
| 31 | TYR | 4,00 | 4,00 | 4,13 | **4,03** | 3,72 | 3,91 | 4,13 | **3,84** |
| 32 | TYR | 3,91 | 3,72 | 3,95 | **3,85** | 3,81 | 3,57 | 3,66 | **3,73** |
| 33 | ASN | 0,11 | 0,70 | 0,28 | **0,35** | -0,14 | -0,45 | 0,42 | **-0,09** |
| 34 | THR | 0,36 | -0,43 | 0,71 | **0,18** | 0,38 | 0,53 | -0,48 | **0,24** |
| 35 | THR | -0,58 | 0,31 | 0,18 | **-0,10** | -0,91 | -0,66 | 0,02 | **-0,68** |
| 36 | LYS | -3,70 | -4,41 | -4,27 | **-4,07** | -3,93 | -4,41 | -3,46 | **-3,94** |
| 37 | GLY | -0,63 | -1,48 | -1,39 | **-1,09** | -0,96 | -0,66 | -0,94 | **-0,90** |
| 38 | GLY | -0,39 | 0,60 | 0,16 | **0,07** | -0,97 | -1,01 | 0,24 | **-0,74** |
| 39 | ARG | -2,64 | -1,91 | -3,43 | **-2,57** | -3,71 | -3,14 | -3,00 | **-3,46** |
| 40 | PHE | 3,15 | 3,51 | 2,13 | **3,03** | 2,91 | 2,36 | 3,67 | **2,95** |
| 41 | VAL | 2,11 | 2,19 | 1,93 | **2,09** | 2,20 | 2,04 | 2,20 | **2,17** |
| 42 | LEU | 3,43 | 3,43 | 3,40 | **3,42** | 2,97 | 3,21 | 3,24 | **3,07** |
| 43 | ALA | 1,93 | 1,93 | 1,93 | **1,93** | 1,93 | 1,93 | 1,93 | **1,93** |
| 44 | LEU | 3,38 | 3,43 | 3,43 | **3,40** | 3,43 | 3,43 | 3,43 | **3,43** |
| 45 | LEU | 3,30 | 3,27 | 3,38 | **3,31** | 3,30 | 3,32 | 3,30 | **3,30** |
| 46 | SER | 0,61 | 0,59 | 0,64 | **0,61** | 0,59 | 0,59 | 0,59 | **0,59** |
| 47 | ASP | -2,82 | -2,34 | -2,71 | **-2,63** | -2,50 | -2,66 | -2,07 | **-2,45** |
| 48 | LEU | 0,96 | 1,57 | 1,32 | **1,25** | 1,38 | 1,59 | 1,49 | **1,44** |
| 49 | GLN | -1,50 | -1,39 | -1,64 | **-1,49** | -1,39 | -1,36 | -1,34 | **-1,37** |
| 50 | ASP | -3,03 | -3,03 | -2,77 | **-2,97** | -3,03 | -2,77 | -3,46 | **-3,06** |
| 51 | LEU | 2,65 | 2,67 | 2,65 | **2,65** | 2,67 | 2,70 | 2,59 | **2,66** |
| 52 | LYS | -1,83 | -1,73 | -1,59 | **-1,74** | -1,88 | -1,92 | -1,02 | **-1,72** |
| 53 | TRP | 3,94 | 3,85 | 3,98 | **3,92** | 3,92 | 3,96 | 3,96 | **3,94** |
| 54 | ALA | 1,92 | 1,93 | 1,93 | **1,92** | 1,93 | 1,93 | 1,93 | **1,93** |
| 55 | ARG | -1,55 | -2,20 | -0,70 | **-1,57** | -1,84 | -1,27 | -1,86 | **-1,73** |
| 56 | PHE | 3,60 | 3,90 | 3,78 | **3,74** | 3,93 | 3,87 | 3,91 | **3,91** |
| 57 | PRO | 1,19 | 1,45 | 0,64 | **1,15** | 1,24 | -0,11 | 0,72 | **0,86** |
| 58 | LYS | -0,98 | -0,93 | -0,93 | **-0,95** | -0,69 | -0,98 | -1,02 | **-0,81** |
| 59 | SER | -2,15 | -0,23 | -2,64 | **-1,62** | -1,39 | -0,99 | -0,55 | **-1,15** |
| 60 | ASP | -3,25 | -2,87 | -3,03 | **-3,07** | -3,14 | -2,98 | -3,25 | **-3,13** |
| 61 | GLY | -0,23 | -0,14 | -0,29 | **-0,21** | -1,04 | -0,21 | -0,74 | **-0,81** |
| 62 | THR | -1,11 | -1,08 | -1,18 | **-1,11** | -1,06 | -1,18 | -1,49 | **-1,17** |
| 63 | GLY | 0,23 | 0,13 | 0,14 | **0,18** | 0,19 | 0,16 | 0,16 | **0,18** |
| 64 | THR | -0,73 | -0,75 | -0,81 | **-0,75** | -0,81 | -0,88 | -0,76 | **-0,81** |
| 65 | ILE | 2,51 | 2,47 | 2,24 | **2,43** | 2,50 | 2,54 | 2,51 | **2,51** |
| 66 | TYR | 2,04 | 2,45 | 2,26 | **2,23** | 2,48 | 2,10 | 2,20 | **2,35** |
| 67 | THR | 0,40 | 0,46 | 0,45 | **0,43** | 0,58 | 0,33 | 0,55 | **0,52** |
| 68 | GLU | -1,93 | -0,95 | -0,44 | **-1,25** | -0,82 | -1,93 | -0,44 | **-0,97** |
| 69 | LEU | 3,43 | 3,43 | 3,43 | **3,43** | 3,40 | 3,43 | 3,43 | **3,41** |
| 70 | GLU | -0,32 | 0,31 | -0,32 | **-0,11** | -0,49 | -0,11 | -0,40 | **-0,40** |
| 71 | PRO | -0,13 | 0,58 | -0,12 | **0,11** | -0,14 | -0,14 | -0,13 | **-0,14** |
| 72 | PRO | 1,15 | 1,43 | 1,12 | **1,24** | 1,58 | 1,18 | 1,43 | **1,47** |
| 73 | CYS | 0,73 | 0,73 | 0,73 | **0,73** | 0,74 | 0,74 | 0,74 | **0,74** |
| 74 | ARG | -4,07 | -4,78 | -4,43 | **-4,39** | -3,78 | -4,07 | -4,43 | **-3,97** |
| 75 | PHE | 3,94 | 3,96 | 3,73 | **3,89** | 3,77 | 3,78 | 3,90 | **3,80** |
| 76 | VAL | 2,06 | 1,06 | 2,23 | **1,76** | 1,99 | 1,90 | 2,31 | **2,03** |
| 77 | THR | 0,65 | 0,60 | 0,60 | **0,62** | 0,70 | 0,71 | 0,65 | **0,69** |
| 78 | ASP | -0,65 | -0,71 | -1,45 | **-0,85** | -0,81 | -0,76 | -1,98 | **-1,03** |
| 79 | THR | 0,28 | 0,36 | 0,27 | **0,30** | 0,36 | 0,41 | 0,36 | **0,37** |
| 80 | PRO | -0,19 | -0,18 | -0,18 | **-0,18** | -0,18 | -0,17 | -0,18 | **-0,18** |
| 81 | LYS | -3,79 | -4,31 | -3,41 | **-3,87** | -4,12 | -4,12 | -4,27 | **-4,15** |
| 82 | GLY | 0,43 | 0,21 | 0,24 | **0,31** | 0,13 | 0,28 | 0,29 | **0,19** |
| 83 | PRO | -0,11 | -0,13 | -0,11 | **-0,12** | 0,45 | -0,13 | 0,54 | **0,35** |
| 84 | LYS | -1,55 | -1,40 | -1,59 | **-1,51** | -0,98 | -1,40 | -1,02 | **-1,07** |
| 85 | VAL | 1,45 | 1,20 | 1,59 | **1,40** | 1,34 | 0,96 | 1,35 | **1,26** |
| 86 | LYS | -0,36 | -0,08 | -0,55 | **-0,31** | -0,03 | 0,21 | -0,08 | **0,01** |
| 87 | TYR | 3,84 | 3,84 | 3,63 | **3,79** | 3,44 | 3,81 | 3,66 | **3,56** |
| 88 | LEU | 3,43 | 3,43 | 3,43 | **3,43** | 3,35 | 3,35 | 3,43 | **3,37** |
| 89 | TYR | 4,13 | 4,13 | 4,13 | **4,13** | 4,13 | 4,13 | 4,13 | **4,13** |
| 90 | PHE | 3,87 | 3,93 | 3,78 | **3,87** | 3,83 | 3,86 | 3,91 | **3,85** |
| 91 | ILE | 3,12 | 3,21 | 3,09 | **3,14** | 3,08 | 3,20 | 3,13 | **3,11** |
| 92 | LYS | -2,04 | -1,36 | -1,26 | **-1,63** | -1,55 | -2,18 | -1,45 | **-1,66** |
| 93 | GLY | -1,51 | -1,28 | -1,51 | **-1,43** | -1,53 | -1,51 | -1,53 | **-1,53** |
| 94 | LEU | 2,62 | 3,43 | 3,32 | **3,05** | 3,24 | 2,51 | 2,54 | **2,96** |
| 95 | ASN | -0,74 | -0,26 | -0,71 | **-0,57** | -0,68 | -1,10 | -0,76 | **-0,78** |
| 96 | ASN | -0,54 | -0,37 | -0,65 | **-0,51** | -0,71 | -0,57 | -0,45 | **-0,63** |
| 97 | LEU | 2,89 | 2,84 | 3,11 | **2,92** | 2,67 | 2,70 | 2,84 | **2,71** |
| 98 | ASN | 0,19 | 0,73 | 0,22 | **0,38** | -0,06 | -0,87 | 0,03 | **-0,21** |
| 99 | ARG | -0,27 | -0,41 | -0,56 | **-0,38** | -0,84 | -0,98 | -1,27 | **-0,95** |
| 100 | GLY | 0,73 | 0,73 | 0,73 | **0,73** | 0,73 | 0,73 | 0,73 | **0,73** |
| 101 | MET | 3,37 | 3,37 | 3,44 | **3,38** | 3,14 | 3,18 | 3,46 | **3,21** |
| 102 | VAL | 2,93 | 2,93 | 2,93 | **2,93** | 2,93 | 2,93 | 2,93 | **2,93** |
| 103 | LEU | 3,43 | 3,43 | 3,21 | **3,38** | 2,89 | 3,16 | 3,13 | **2,99** |
| 104 | GLY | 0,73 | 0,73 | 0,70 | **0,72** | 0,73 | 0,71 | 0,73 | **0,73** |
| 105 | SER | 0,70 | 0,59 | 0,67 | **0,66** | 0,67 | 0,64 | 0,70 | **0,67** |
| 106 | LEU | 3,40 | 3,43 | 3,40 | **3,41** | 3,43 | 3,35 | 3,43 | **3,41** |
| 107 | ALA | 1,79 | 1,84 | 0,96 | **1,61** | 1,85 | 1,78 | 1,67 | **1,80** |
| 108 | ALA | 1,49 | 0,76 | 0,48 | **1,01** | 1,32 | 1,32 | 0,48 | **1,16** |
| 109 | THR | -0,07 | -0,13 | 0,07 | **-0,06** | -0,12 | -0,18 | 0,12 | **-0,09** |
| 110 | VAL | 2,24 | 2,49 | 2,43 | **2,37** | 2,48 | 2,12 | 2,58 | **2,43** |
| 111 | ARG | -4,27 | -4,05 | -4,55 | **-4,26** | -3,48 | -3,93 | -5,12 | **-3,89** |
| 112 | LEU | 3,35 | 3,21 | 2,94 | **3,20** | 2,70 | 2,70 | 3,40 | **2,84** |
| 113 | GLN | -1,08 | -2,41 | -1,00 | **-1,51** | -1,76 | -1,40 | -0,84 | **-1,51** |
